# The Significance of Duration of Exposure and Circulation of Fresh Air in SARS-CoV-2 Transmission Among Healthcare Workers

**DOI:** 10.3389/fmed.2021.664297

**Published:** 2021-06-24

**Authors:** Vasiliki Vlacha, Gavriela Feketea, Athanasia Petropoulou, Sebastian Daniel Trancá

**Affiliations:** ^1^Department of Early Years Learning and Care, University of Ioannina, Ioannina, Greece; ^2^PhD School, Iuliu Hatieganu University of Medicine and Pharmacy, Cluj-Napoca, Romania; ^3^Pediatric Department, Amaliada Hospital Unit, General Hospital of Ilia, Amaliada, Greece; ^4^Nursing Service, Amaliada Hospital Unit, General Hospital of Ilia, Amaliada, Greece; ^5^Department of Anesthesia and Intensive Care II, Iuliu Hatieganu University of Medicine and Pharmacy, Cluj-Napoca, Romania

**Keywords:** COVID-19, SARS-CoV-2 transmission, health care worker, health care facilities, safety measures

## Abstract

**Background:** The true risk of infection after exposure to SARS-CoV-2 of healthcare workers (HCWs) in the workplace has not yet been established. This descriptive study analyzes the exposure characteristics of HCWs to SARS-CoV-2.

**Methods:** In March 2020, at the beginning of the pandemic, a total of 58 HCWs in a regional hospital in Greece were exposed to three patients with symptomatic SARS-CoV-2 infection. These three index cases had taken part in an 8-day religious tour, during which 52 travelers spent 10 h every day in a tour bus. A study was made of the circumstances of the hospital exposure.

**Results:** Of the 52 travelers in the bus, 48 contracted SARS-CoV2. None of the 58 HCW contacts developed symptoms related to COVID-19, although, 43% were exposed to a SARS-CoV-2 infected patient for more than 15 min, and 74% were within a distance of <1 m, and half of the contacts were not wearing a surgical mask. Additional information was that 63% of the contacts were exposed in a room sized more than 15 m^2^, and in more than 80% of cases, the window or the door to the room was open during their exposure. In about one-third of the exposure events, the HCW contacts were not wearing a mask and were at a distance of <1 m, and just under half of them were exposed for more than 15 min. One-fourth of the contacts underwent RT-PCR testing, and 11% IgG/IgM antibody testing for SARS-CoV-2, all of which were negative. All observed quarantine at home for 14 days.

**Conclusion:** This observational study was made before the extent of the SARS-CoV-2 became apparent, and before routine preventive measures were observed by all HCWs. Comparing the conditions of exposure in the two different settings (bus vs. regional health facility), it is apparent that the duration of exposure and the small, enclosed nature of the bus are the distinguishing factors. In the healthcare setting, the elimination of both factors and the implementation of additional measures protected the exposed HCWs from contracting SARS-CoV-2 infection.

## Introduction

As the spread of COVID-19 is changing rapidly, there are still many unknown factors regarding its transmissibility. Recently, details of the aerogenic transmission of SARS-CoV-2 have been documented by several researchers (1).

The combination of several factors may affect the transmissibility of SARS-CoV-2, including distance (2, 3), viral load (4), duration of exposure (5, 6), and mask wearing (1). The World Health Organization (WHO) (7) and the US Centers for Disease Control and Prevention (CDC) (8) have recommended specific protection measures for work places. In addition, mutations in spike protein cause increased infectivity (9).

Healthcare workers (HCWs) are in the front line of fighting the pandemic (10). The published findings on infections and deaths among exposed HCWs are devastating (11, 12). Personal protective equipment (PPE) including mask, gloves, and non-woolen robes are recommended by several public health authorities (7, 13, 14).

In this observational study, we analyzed the characteristics of the personnel exposed to three patients with COVID-19 in a regional hospital in Greece at the very beginning of the pandemic, when no other cases had been identified in the community, and before the policies regarding the protection of the HCWs from SARS-CoV-2 had been broadly implemented.

## Methods

### The Patients

Patient No 1 was 66-year-old man, who had just returned to Greece from an organized religious group trip to holy sites in the Middle East. He became ill the day after his arrival back in Greece, with a sore throat, fever of 38°C, and myalgia. Two days later, his fever rose to 39.2°C, at which stage he attended the emergency department (ED) of the regional hospital. He was hospitalized for 2 days, then, on diagnosis of SARS-CoV-2, he was transferred to a COVID-19 unit in a tertiary hospital, where he finally died on day 15 of his illness. His wife, who had also been on the religious trip, was diagnosed with COVID-19 3 days after this patient's admission.

Patient No 2 was a 45-year-old man who had been on the same religious trip. He attended the ED with fever and cough and was admitted with pneumonia. He was transferred to the tertiary hospital and remained hospitalized for 21 days, but recovered. This patient had attended a funeral the day prior to his admission, and several other funeral attendees were subsequently diagnosed with COVID-19. The brother of patient No 2 also developed COVID-19, as they met shortly after the return from the religious tour.

Patient No 3 was 35-year-old woman, who was an administrative officer in the regional hospital, and who also had been on the religious trip. She developed fever and myalgia 3 days prior to returning to work. She was informed about the spread of SARS-CoV-2 during the religious trip after being back at work for 2 days, at which time she had minimal symptoms, with no cough. She was not hospitalized but remained in isolation at home.

Table 1 summarizes the clinical characteristics of the three patients (index cases) with COVID-19 diagnosed at the regional hospital. All three individuals had been symptomatic for a minimum of 2 days prior to their hospital visit. Two of them had cough, were diagnosed with pneumonia, and stayed hospitalized in the regional hospital for 2–4 days prior to transfer to a tertiary Medical Center due to their clinical deterioration. None of the patients had gastrointestinal symptoms.

**Table 1:** The clinical characteristics of the first three patients with COVID-19 diagnosed in a Greek regional hospital.

**Characteristics**	**Patient 1**	**Patient 2**	**Patient 3**
Estimated time from exposure to onset of symptoms (days)	7	7	6
Symptoms—duration (days)			
• Cough	3	6	No
• Fever	3	3	2
• Myalgia	3	No	3
• Fatigue	3	4	5
• Headache	3	No	No
• Sore throat	3	2	2
• Loss of smell	No	No	No
• Shortness of breath (days)	3	2	No
• Gastrointestinal symptoms	No	No	No
• Hospitalization	Yes	Yes	No
Duration of symptoms prior to visit to regional hospital (days)	2	6	3
Duration of hospitalization in regional hospital (days)	3	4	No hospitalization
Transfer to a tertiary hospital	Yes	Yes	No hospitalization
Lung involvement	Pneumonia	Pneumonia	No lung involvement
Outcome	Death	Cure	Cure

These three patients were the index cases of an outbreak of COVID-19 among a group of 52 Greeks who had participated in an organized religious bus tour to holy sites in the Middle East. They were riding in the bus for approximately 10 h per day for a total of 8 days. Of the 52 individuals in the group, 48 tested SARS-CoV-2 positive, and 2 finally died of complications of the disease.

All contacts of the three index cases who were identified, including HCWs in the hospital, completed a questionnaire regarding their exposure to the infected person. In this way, we gathered information relating to the duration of the exposure of the HCWs, their distance from the index case, the size of the room, whether the windows/door were open or closed, the age of the exposed person, the occupation, and the PPE that was used, if any. The protective measures introduced by the healthcare facility to medical personnel at that time was in accordance to WHO interim guidelines (2/27/2020) for suspected cases. Those included, application of surgical mask and gloves, provide adequate space to allow at least 1 m distance, limit the time of exposure, and open air ventilation. However, the index of suspicion was low, as no other COVID-19 cases were identified in Greece up that point (15). Those measures were suggested but not mandated since no COVID-19 cases were identified in Greece up to that point. RT-PCR for SARS-Co-V-2 (VIASURE, CerTest Biotec) was performed by nasopharyngeal swabs on all the HCWs who were exposed at a distance of <1 m from the symptomatic patients. All exposed hospital personnel remained on home isolation for 14 days. They were instructed to self-assess and report symptoms related to COVID-19. Eight weeks after the exposure, the contacts were questioned again about their clinical status and were tested for SARS-CoV-2 antibodies using Abbott SARS-CoV-2 IgM and IgG performed on the Abbott automated analyzer.

## Results

A total of 58 contacts, each with one of the three index cases, were identified among the hospital HCWs. None of the exposed personnel developed symptoms.

The median age of the HCW contacts was 47.74 years, ranging from 25 to 60 years, with a predominance of female staff (72.42%). Of the exposed personnel, 17 (29.3%) were physicians, 20 (34.5%) were registered nurses, and 12 (20.9%) were administrative staff.

Regarding the duration of exposure, 33/58 (56.9%) had remained in contact with one of the index cases for <15 min, 16 (27.6%) for 15 min to 2 h, 4 (8.6%) for 2–4 h, and 5 (8.6%) for more than 4 h. Regarding the distance from the index case, 43 (74.1%) of the contacts were within 1 m of an index case, 13 (22.4%) were at a distance of 1–3 m, and 2 contacts (3.4%) were at a distance more than 3 m.

The size of the room in which the contact had been made was evaluated. Exposure in a small room of less 15 m^2^ was reported by 21/58 (36.2%) contacts, and 32/58 (55.2%) in a room of 15–30 m^2^; overall, 63.8% of the contacts were exposed in a room sized more than 15 m^2^.

**Figure 1:**
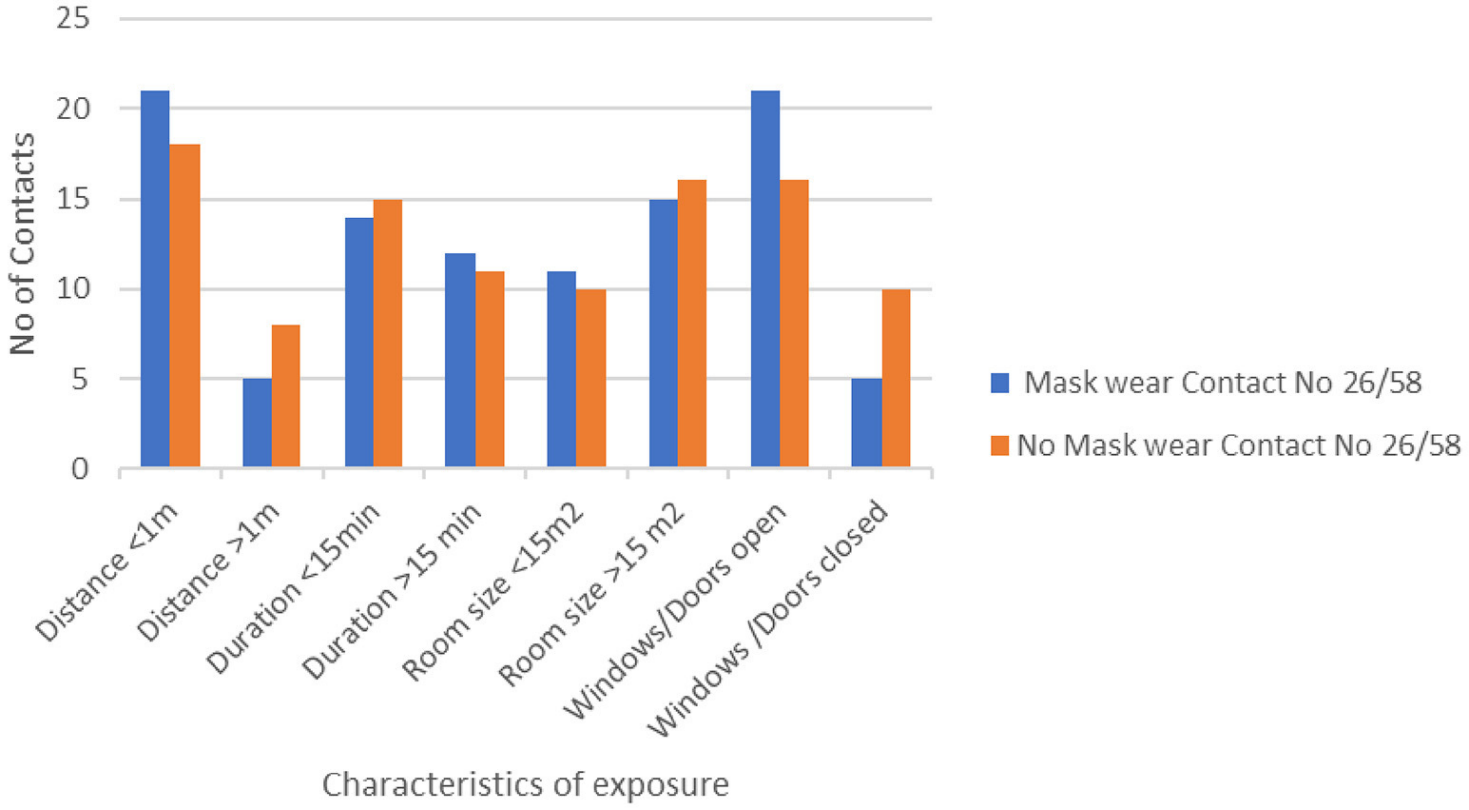
Characteristics of the contact of healthcare workers (*N* = 58) with three patients with SARS-CoV-2, with and without masks.

Full PPE was not worn by any of the contacts (the events took place before the extent of the COVID-19 threat was apparent). Some of the exposed hospital staff were using surgical masks, surgical gloves, and/or a cotton robe during exposure. Almost half (44.8%) of the exposed staff wore a surgical mask during the exposure, while 10.4% did not remember. Of the 16 administrative staff members exposed to patient No 3 (their colleague), only 2 (12.5%) wore a surgical mask. They were at a distance of 1–3 m, and their exposure lasted for <15 min. Figure 1 shows a comparison of the contact characteristics of the contacts with and without masks.

Medical and nursing care was administered by 24 of the 58 contacts (41.4%). Of these 24 contacts, 17 (70.8%) wore a surgical mask, 5 of the 24 (20.8%) did not wear a mask, and 2 did not remember. All five contacts who did not wear a mask were at a distance of <1 m from an index case, and three of them for more than 15 min, one of the three in a room sized more than 15 m^2^. Of the 58 contacts, 36 (62%) wore gloves, with 24 of the 36 (66.7%) performing medical or nursing procedures. In addition, 22 of the 24 (91.7%) who performed medical or nursing procedures wore a cotton gown.

**Figure 2:**
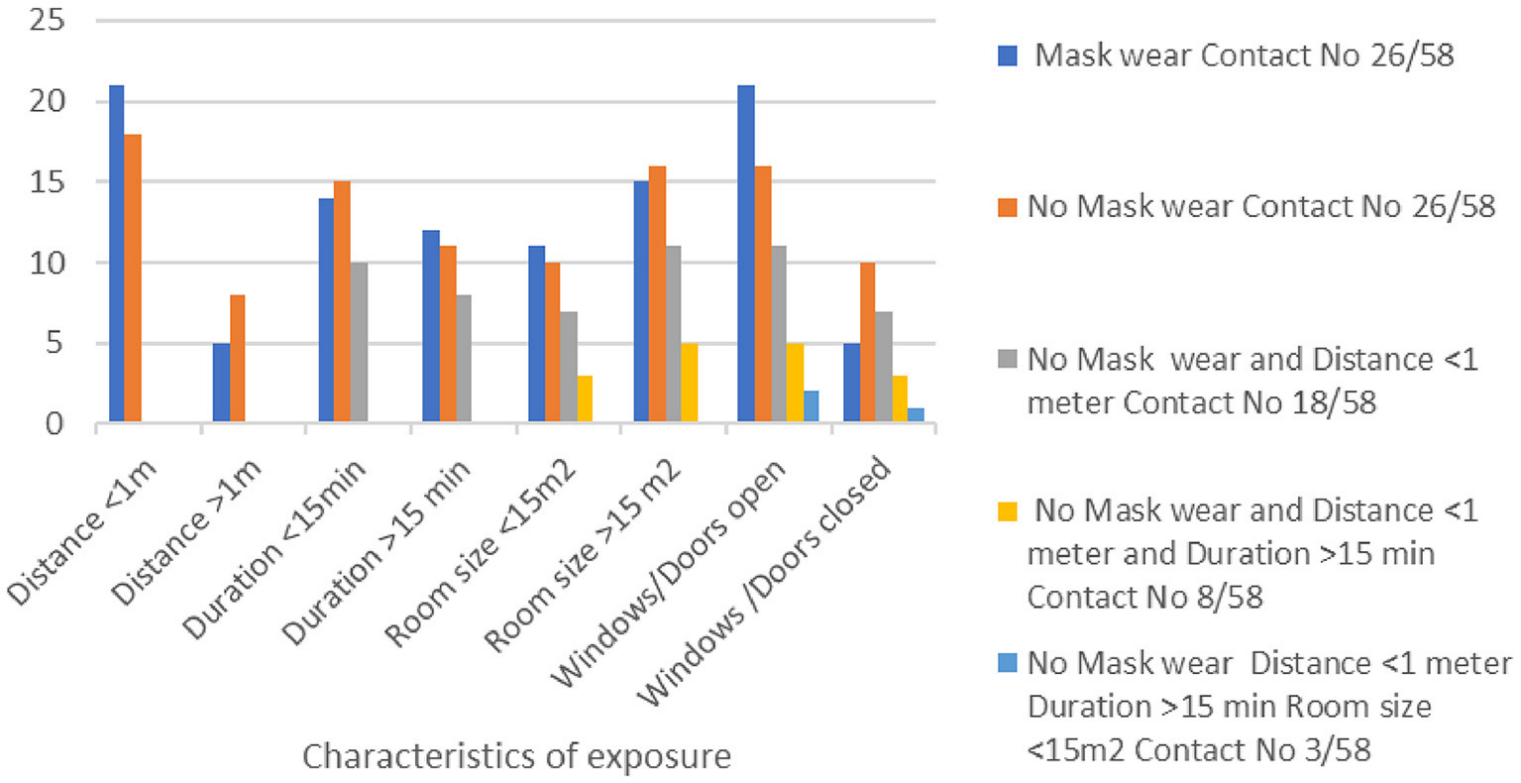
Characteristics of the contact of healthcare workers (*N* = 58) with three patients with SARS-CoV-2 in a regional hospital in Greece at the beginning of the COVID-19 pandemic.

We also investigated the question of circulation of fresh air in the room occupied by the index cases, by recording whether the windows/door remained open or closed during the exposure. An open door was reported by 50/58 contacts (86.2%), and open windows by 42/58 (72.4%). Comparisons among all the contacts based on the characteristics of their exposure are shown in Figure 2.

RT-PCR SARS-CoV-2 tests were performed in 14/58 contacts (24.1%), antibody tests for SARS-CoV-2 in 11/58 contacts (19%), and 8/58 (13.8%) had both tests done. All the tests were reported negative.

On analyzing the data collected from the total group of 58 contacts, a highly exposed subgroup was identified, consisting of 18 contacts (31%) who did not wear a mask and had been closer than 1 m to an index case. Of these, 8/18 (44.4%) were exposed for more than 15 min, and 4/18 (22.2%) were exposed for more than 4 h. In five of the eight cases of close contact (62.5%), the windows had been open, and in two of the three with the windows closed, the door was open. In addition, 8/21 (38%) contacts who reported being in a small room with an index case said that the windows were closed, but in 4/8 (50%) cases, the doors were open. Only three of the contacts reported being in a small room at a distance <1 m from the index case with both doors and window closed, two wearing a surgical mask, but one without a mask. Two others did not remember whether they were wearing a mask or not. One physician who came in contact with patient No 1 did not wear a mask and was at a distance of <1 m from the patient for more than 15 min with windows and door closed, in spite of which she tested negative for SARS-CoV-2 antibodies and she never developed any symptoms related to COVID-19.

RT-PCR SARS-CoV-2 was performed in 6/18 (33.3%) highly exposed contacts and one had an antibody test, all of which were reported negative.

In total, 42 hospital staff (72.4%) came into contact with the symptomatic patients No 1 and No 2, of whom 37 were at a distance of <1 m, and 17/42 (40.5%) for a duration of more than 15 min. In addition, 12/42 contacts (28.6%) were not wearing a mask and were at a distance of <1 m; of these, four had a negative RT-PCR test for SARS-CoV-2 and 3 had a negative SARS-CoV-2 antibody test.

The case of patient No 3 was different from the other two, as she was working in the administration department, and her hospital contacts were mainly colleagues, 16 in number. The duration of the contact was more than 15 min in 50%, and 6/16 (37.5%) were at a distance of <1 m, four of them for more than 4 h. Most (87.5%) of these contacts did not wear a surgical mask, but in 13/16, the windows were open during the exposure, and the door was open in all cases. The size of the room was more than 15 m^2^ in 8/16 cases. All the personnel exposed at a distance of <1 m said that they had the windows open, but they did not wear a surgical mask.

The characteristics of the contacts with patients No 1 and 2 with severe symptoms and patient No 3 with mild symptoms are shown in Table 2.

**Table 2:** Exposure characteristics of hospital staff (*N* = 58) who came into contact with three patients with SARS-CoV-2 (patients 1 and 2 with severe, and patient 3 with mild symptoms).

	**Exposure to patient no 1 and 2**	**Exposure to patient no 3**
	**Total number of contacts: 42**	**Total number of contacts: 16**
**Exposure characteristics**	**Number of contacts (%)**	**Number of contacts (%)**
**Surgical masks**		
Yes	24 (57.2%)	2 (12.5%)
No	12 (28.5%)	2 (12.5%)
Don't remember	6 (14.3 %)	12(75%)
**Surgical gloves**		
Yes	32 (76.2%)	6 (37.5%)
No	8 (19.0%)	9 (56.3%)
Don't remember	2 (4.8%)	1 (6.2%)
**Distance**		
<1 m	37 (88%)	6 (37.5%)
1–3 m	4 (9.6%)	9 (56.3%)
>3 m	1 (2.4%)	1 (6.2%)
**Duration of contact**		
<15 min	25 (59.5%)	8 (50%)
15 min−2 h	13 (30.9%)	3 (18.8%)
2–4 h	3 (7.1%)	1 (6.2%)
>4 h	1 (2.5%)	4 (25%)
**Room size**		
<15 m^2^	13 (31.0%)	8 (50%)
15–30 m^2^	25 (59.5%)	8 (50%)
>30 m^2^	4 (9.5%)	
**Windows**		
Open	29 (69.0%)	13 (81.3%)
Closed	13 (31.0%)	3 (18.7%)
**Doors**		
Open	34 (81.0%)	16 (100%)
Closed	8 (19.0%)	
**RT-PCR**		
Yes	12 (28.6%)	2 (12.5%)
No	30 (71.4%)	14 (87.5%)
**SARS-CoV2 antibodies**		
Yes	8 (19%)	2 (12.5%)
No	34 (81%)	14 (87.5%)

## Discussion

This study represented a unique opportunity to analyze the exposure of HCWs to patients with SARS-CoV-2 in a Greek regional healthcare setting at the beginning of the COVD-19 pandemic at a time when there were no recognized community exposures, and the relevant safety measures had not yet been fully introduced.

The three most important components of the recommended safety measures, namely, wearing of a mask, distance from the index case, and duration of exposure, were all significantly compromised. In spite of this, the final outcome of all the exposed HCWs was to remain asymptomatic during the 8-week follow-up in isolation, which was implemented when the diagnosis of SARS-CoV-2 was made (16, 17).

This group of 58 contacts with three index cases of SARS-CoV-2 recorded several specific high-risk factors; two-thirds of the contacts were at a distance of <1 m from the index case, half were not wearing a surgical mask during their exposure, and two-fifths of them remained in contact with an index case for more than 15 min. Several other factors appear to have protected them from contracting the virus.

When comparing the bus riders (48/52 got infected) and the HCWs (none got infected), it seems that the most distinguishing differences are the length of contact and the small and enclosed nature of the bus. The high viral transmissibility in small, confined spaces has been shown in a study performed by Kasper et al. (18) in a nuclear-powered aircraft carrier.

Although, this incident took place before COVID-19 regulations were fully implemented, and there was initially no reason to suspect that patients 1 and 2 posed a special threat, a significant proportion (70.8%) of the personnel who performed a medical or nursing procedure on these patients reported wearing a mask during contact with them. Additional protective equipment such as a cotton robe and surgical gloves were also worn in some cases, but the HCWs did not perform aerosolized procedures (1). None of the exposed HCWs developed symptoms, even though 13.8% did not wear a mask and were within close distance for more than 15 min, and three were in a small room within a distance of <1 m of the index case for more than 15 min with doors and window closed, one without a mask (19, 20). Patients 1 and 2 were symptomatic on admission, with cough, and a diagnosis of pneumonia was made. They both required medical intervention, with 2–4 days of hospitalization before transfer to the tertiary center, and one subsequently died. About 25% of the HCW wore no mask and were within <1 m while examining or administering treatment, and 25% of those were exposed for more than 15 min.

Regarding the administrative staff exposed in their workplace to patient 3, their colleague, only a small number of the administrative officers wore a mask, but most were at a distance of more than 1 m and the duration of exposure was <15 min.

The three index cases presented in this study contracted SARS-CoV-2 while traveling on the same religious bus tour where 48/52 tourists in the group were infected. They were riding in the bus for a total of 8 days, in close contact with each other, for approximately 10 h per day, with breaks every 2–3 h when the bus was naturally ventilated. This implies that the transmissibility of the specific viral strain was high, at least in the contained environment of a tour bus, with lengthy exposure. In the hospital environment where the factors of the enclosed space and extended duration of contact were eliminated, the HCWs did not contact the virus.

This is among the first known reports where occupational transmission of SARS-CoV-2 has not been recorded, despite the fact that the HCWs were not using contact, droplet, or airborne precautions when in contact with an infected patient. The results of this study do not negate the need for application of PPE for protection of HCWs, as has been suggested on previous studies (12, 21), but they indicate the value of additional attention to environmental measures to augment the protection of this vulnerable group of first line workers. Those measures should be reinforced in the face of the merge of new SARS-CoV-2 variants with increased infectivity (22).

Nguyen and colleagues conducted a prospective cohort study, using the COVID Symptom Study smartphone application, and found that adequate supplies of PPE did not completely mitigate the infection rate in high-risk exposures for HCWs (21).

Our study has certain limitations, including the inadequate number of RT-PCR tests for SARS-CoV-2 in the exposed personnel, and the absence of infection in the HCW contacts was evaluated according to the reported absence of symptoms. This study was conducted in the ED and the regular hospital in-patient department of a small regional hospital, and not in an intensive care unit. No aerosolized procedures were performed on the index cases. As noted above, the HCWs were exposed to only one infected patient. The air circulation and filtration in the bus were not evaluated in this study, which was restricted to hospital exposure.

## Conclusion

Our findings point out the high transmissibility of the virus in lengthy exposure and in a small, enclosed place of a bus. On the other hand, in the healthcare facility where those factors were eliminated, and further measures were in place, the HCWs were protected. Additional studies are needed to be performed on the air circulation of buses where a high infection rate was seen.

## Data Availability Statement

The raw data supporting the conclusions of this article will be made available by the authors, without undue reservation.

## Ethics Statement

The studies involving human participants were reviewed and approved by Hospital ethics and scientific committee of Amaliada Hospital Unit, General Hospital of Ilia, Amaliada, Greece. The patients/participants provided their written informed consent to participate in this study. Written informed consent was obtained from the individual(s) for the publication of any potentially identifiable images or data included in this article.

## Author Contributions

VV, GF, and AP: concept and design of the study. GF and AP: acquisition of data. VV, GF, and ST: analysis and interpretation of data and drafting the article. All authors have approved the final version to be submitted.

## Conflict of Interest

The authors declare that the research was conducted in the absence of any commercial or financial relationships that could be construed as a potential conflict of interest.
